# Harmful Alcohol Use Among Healthcare Workers at the Beginning of the COVID-19 Pandemic in Kenya

**DOI:** 10.3389/fpsyt.2022.821610

**Published:** 2022-02-28

**Authors:** Florence Jaguga, Edith Kamaru Kwobah, Ann Mwangi, Kirtika Patel, Thomas Mwogi, Robert Kiptoo, Lukoye Atwoli

**Affiliations:** ^1^Department of Mental Health, Moi Teaching & Referral Hospital, Eldoret, Kenya; ^2^School of Science and Aerospace Studies, Moi University, Eldoret, Kenya; ^3^Department of Pathology, School of Medicine, Moi University, Eldoret, Kenya; ^4^Department of Internal Medicine, Moi Teaching & Referral Hospital, Eldoret, Kenya; ^5^Department of Mental Health and Behavioral Sciences, School of Medicine, Moi University, Eldoret, Kenya; ^6^Brain and Mind Institute and the Department of Internal Medicine, Medical College East Africa, Aga Khan University, Nairobi, Kenya

**Keywords:** alcohol, healthcare, workers, COVID-19, Kenya

## Abstract

**Background:**

Healthcare workers play a key role in responding to pandemics like the on-going COVID-19 one. Harmful alcohol use among them could result in inefficiencies in health service delivery. This is particularly concerning in sub-Saharan Africa where the health workforce is already constrained. The aim of this study is to document the burden and correlates of harmful alcohol use among healthcare workers at the beginning of the COVID-19 pandemic in Kenya with the aim of informing policy and practice.

**Methods:**

This study was a cross-sectional analysis of data obtained from a parent online survey that investigated the burden and factors associated with mental disorders among healthcare workers during the COVID-19 pandemic in Kenya. We analyzed data obtained from a sub-population of 887 participants who completed the Alcohol Use Disorder Identification Test questionnaire. We used descriptive statistics to summarize the socio-demographic characteristics of the participants and multivariate analysis to determine the factors associated with harmful alcohol use.

**Results:**

Three hundred and eighty nine (43.9%) participants reported harmful alcohol use. The factors significantly associated with increased odds of endorsing harmful alcohol use were: being male (AOR = 1.56; 95% CI = 1.14, 2.14; *p* = 0.006), being unmarried (AOR = 2.06; 95% CI = 1.48, 2.89; *p* < 0.001), having 11-20 years of experience as compared to having 20+ years of experience (AOR = 1.91; 95% CI = 1.18, 3.12; *p* = 0.009), and being a specialist (AOR = 2.78; CI = 1.64, 4.78; *p* = < 0.001) or doctor (AOR = 2.82; 95% CI = 1.74, 4.63; *p* < 0.001) as compared to being a nurse.

**Conclusions:**

A high proportion of healthcare workers reported harmful alcohol use at the beginning of the COVID-19 pandemic in Kenya. Males, the unmarried, those with 11–20 years of experience in the health field, doctors and specialists, were more likely to report harmful alcohol use. These findings highlight the need to institute interventions for harmful alcohol use targeting these groups of healthcare workers in Kenya during the COVID-19 pandemic in order to optimize functioning of the available workforce.

## Introduction

Healthcare workers play a critical role in responding to pandemics like the coronavirus disease of 2019 (COVID-19) (1). In addition to being involved in direct patient care, they are expected to educate the public and patients on infection prevention practices, conduct public health reporting, and at the same time strictly adhere to the established occupational health and safety procedures (2). Because of this central role in responding to the COVID-19 pandemic, the World Health Organization (WHO) recommends that a number of interventions (individual, organizational and systems level) are put in place in order to preserve, manage and optimize the health workforce during the pandemic (1). This is particularly important in resource-limited settings like Kenya, where the healthcare workforce is already constrained. For example as of 2017, Kenya had a total of 90,000 physicians and nurses (3), translating to a density of 2 skilled healthcare workers per 1,000 population against the minimum recommended 4.45 (4).

At the individual level, the WHO recommends that interventions that safeguard the mental health of healthcare workers are implemented (1). Health care workers are highly vulnerable to psychological distress during pandemics because they often have direct contact with infected persons, face increased workload, and are constantly exposed to potentially traumatic events in the course of disease outbreaks. Indeed studies conducted during the COVID-19 pandemic indicate a high psychological impact on health care workers including depression, anxiety and post- traumatic stress (5).

Harmful alcohol use is a particularly important mental health problem that could impact the availability and capacity of healthcare workers to deliver health services during the pandemic (6). Interventions targeting harmful alcohol use among healthcare workers during the COVID-19 pandemic need to be prioritized for two main reasons: Firstly harmful alcohol use is associated with reduced performance and productivity in the workplace emanating from associated ill health and cognitive impairments (6). Secondly, COVID-19 puts healthcare workers at risk of increased alcohol use as a result of maladaptive attempts at coping with the high levels of burnout, depression and anxiety associated with the pandemic (7–9). Indeed studies conducted among healthcare workers in Europe and the United States (US) have reported an increase in alcohol consumption after the onset of the COVID-19 pandemic (10–12).

The need for evidence on the prevalence of harmful alcohol use among healthcare workers during the COVID-19 pandemic is pressing, particularly in sub-Saharan Africa, which has one of the most significant healthcare worker shortages globally (13). Unfortunately, little has been done to explore the burden of harmful alcohol use in that region during the COVID-19 pandemic. Available work has mostly been conducted in western settings (14–16). Rates of problem drinking among healthcare workers during the COVID-19 pandemic have been reported as 7% in the United Kingdom (UK) (15), and 42.6% in the United States (US) (14) based on AUDIT-C cut-off scores of >7 and >4 respectively. One study conducted in Ethiopia reported the prevalence rate of alcohol use, once or more in the past 3 months, as 40.2% among medical and non-medical healthcare workers during the pandemic (17).

The aim of the present study is to report on the prevalence and factors associated with harmful alcohol use among healthcare workers at the beginning of the COVID-19 pandemic in Kenya. The first case of COVID-19 was confirmed in Kenya on 12th March 2020 (18). This study was conducted between April 27th and June 5th 2020, two months after the onset of the pandemic in Kenya. During the study period, new confirmed cases rose from 15 (19) to 124 (20) while COVID-19 related deaths increased from 21 (19) to 78 (20). Public health measures included targeted testing, travel restrictions, training health providers on COVID-19 and its management, and educating the public on preventive measures (19). Health care workers faced a number of challenges at that time including inadequate personal protective equipment; lack of quarantine facilities after shifts in the isolation wards and this exposed their families to the risk of contracting COVID-19; and hostile clients (20).

The findings of our study could be useful in implementing alcohol treatment and prevention interventions aimed at preserving and optimizing the health workforce, as well as maintaining health care worker well-being during the COVID-19 pandemic in Kenya and in other settings in sub-Saharan Africa.

## Materials and Methods

Data used for this study were derived from a parent online survey investigating the prevalence and correlates of mental disorders among healthcare workers (nurses, doctors, clinical officers^1^ and public health officers^2^ during the early phase of the COVID-19 pandemic in Kenya (7). These cadres of healthcare workers were directly involved in preventive, promotive and treatment activities during the covid-19 pandemic in Kenya. Eligible participants for the online survey were trained health professionals working in healthcare at the beginning of the pandemic in the country. Health professionals working outside hospital settings, e.g., insurance companies were excluded. A virtual snowball convenient sampling technique was utilized to recruit participants. We used this mode of sampling because there was no database for healthcare workers' contacts which would allow for randomization. In total, 1,190 healthcare workers consented to participate in the survey. Of these, 957 completed at least one or more of the questionnaires.

The survey instrument was programmed into Redcap (Research Electronic Data Capture) (21), a secure, web-based software platform designed to support data capture for research studies. The survey was comprised of the following questionnaires:

**A researcher designed questionnaire** was used for collecting socio-demographic data including age, sex (male/female), marital status (married/not married), cadre (doctor/nurse/specialist/other), type of facility (public/private), contact with COVID-19 patients (yes/no), years of experience in healthcare (0–10, 11–20, 20+), and history of a chronic medical condition (yes/no).

The primary outcome for this study was harmful alcohol use. This was measured using the **Alcohol Use Disorder Identification Test (AUDIT)** which examined past year alcohol use and consisted of 10 questions with total scores ranging from 0 to 40 (22). A score of 8 and above was considered harmful alcohol use for our study (22). The AUDIT has been used among adults in Kenya (23).

Depression was measured using the **Patient Health Questionnaire-9 (PHQ-9)** (25). It is a 9-item self-report instrument with total scores ranging from 0 to 27. The PHQ-9 examined for depressive symptoms over the past 2 week period. A score of 0–4 was considered no depression, 5–9 mild depression, 10–14 moderate depression, 15–19 moderately severe depression, and 20–27 severe depression (25). The PHQ-9 has excellent reliability and validity. The PHQ-9 has been validated among adults in Kenya (26).

**Generalized Anxiety Disorder (GAD)** was measured using the GAD-7 scale, a seven item self-report instrument (27). The GAD-7 was used to examine for generalized anxiety symptoms among the participants over the past 2 week period. Total scores ranged from 0 to 21. A score of 0–4 was considered no GAD, 5–9 mild GAD, 10–14 moderate GAD, and 15–21 severe GAD for our study (27). The GAD-7 has been validated in Kenya (28).

The **Pittsburgh Sleep Quality Index (PSQI)** (29) was used to assess for sleep quality. It is a self-rated questionnaire which assesses for sleep quality and disturbances over a 1-month time interval. Nineteen individual items generate seven “component” scores: subjective sleep quality, sleep latency, sleep duration, habitual sleep efficiency, sleep disturbances, use of sleeping medication, and daytime dysfunction. The sum of scores for these seven components yields one global score. A score of 5 and above indicated poor quality sleep for our study. Such a score has a sensitivity of 89.6% and specificity of 86.5% in distinguishing good and poor quality of sleep (29). The tool has been used among adults in Kenya (30).

The online survey was sent to healthcare workers using various networks on Facebook, WhatsApp and E-mail. A weekly reminder to participate was sent through the various platforms between April 27th and June 5th 2020. The healthcare workers were requested to respond to the survey and share with their colleagues. A track of responses was kept using the Redcap software until there were no new responses for a period of 2 weeks, after which the survey was closed. The detailed methods for the parent study have been published elsewhere (7).

### Statistical Analysis

The analysis for this study is based on data obtained from a sub-population of 887 participants who completed the AUDIT questionnaire (22). Regarding the non-respondents, there were 233 who consented but did not complete the socio demographic part of the questionnaire hence we would not have their demographic data to include in Table 1. Thus the only ones who did not respond to the AUDIT were 70 i.e., 7.3% of those with demographic data. This is low and excluding them would therefore not bias the results.

**Table 1:** Socio-demographic characteristics of the participants.

**Variable**		***N* (%)**
Age in years	<35	431 (48.6)
	≥35	456 (51.4)
Gender	Male	403 (45.4)
	Female	484 (54.6)
Marital status	Married	579 (65.3)
	Not married	308 (34.7)
Years of experience in healthcare	0-10	512 (57.7)
	11–20	219 (24.7)
	20+	156 (17.6)
Cadre	Doctor	354 (39.9)
	Nurse	167 (18.8)
	Other	216 (24.4)
	Specialist	149 (16.8)
Type of facility	Public	621 (70.0)
	Private	266 (30.0)
Have a chronic medical condition	Yes	202 (22.8)
	No	685 (77.2)
Contact with COVID-19 patients	Yes	212 (23.9)
	No	675 (76.1)

Descriptive statistics were used to summarize the socio-demographic characteristics of the participants. Chi square test was used in the bivariate analysis, to assess for the association between harmful alcohol use and socio-demographic factors as well as the association between harmful alcohol use and depression, generalized anxiety and sleep quality. Significant variables were subjected to the multivariate logistic regression analysis and presented as adjusted odds ratios (AORs) and 95% Confidence Intervals (CIs).

Since the percentage of completion for each questionnaire was not the same. The regression analysis was based on the complete case analysis of those who had data on the variables included in the regression model. Data analysis was performed using R Core Team (31). In all analyses a *p*-value < 0.05 was considered significant.

## Results

### Socio-Demographic Characteristics of Participants

Most of the participants were aged 35 years and above (51.4%); most were female (54.6%); most worked in public health facilities (70.0%); and most had 10 years or less of experience in healthcare (57.7%). Less than one third of the participants (24.0%) had come into contact with a patient diagnosed with COVID-19. Forty percent of the participants were doctors, 18.8% were nurses, 16.8% were specialists, and 24.4% belonged to other cadres (Table 1).

### Mental Health Characteristics of the Participants

Out of the 887 participants who responded to the AUDIT questionnaire, 858 (96.7%) completed the PHQ-9; 807 (91.0%) completed the GAD-7, and 772 (87.0%) completed the PSQI. All the participants (100%) who completed the PHQ-9 endorsed some level of depression. Thirty six percent of those who completed the GAD-7 reported some level of GAD, while poor sleep quality was endorsed by 24.5% of those who completed the PSQ-I (Table 2).

**Table 2:** Mental health characteristics of the participants who completed the AUDIT.

**Depression^**a**^ (*N* = 858^**b**^)**	***n* (%)**
Mild	581 (67.7)
Moderate	144 (16.8)
Severe	133 (15.5)
**GAD**^**c**^ **(*****N*** **=** **807**^**b**^**)**	
None	516 (64.0)
Mild/Moderate	232 (28.7)
Severe	59 (7.3)
**Sleep quality**^**d**^ **(N** **=** **772**^**b**^**)**	
Poor quality of sleep	189 (24.5)
Good quality of sleep	583 (75.5)

a *A score of 5–9 mild depression, 10–14 moderate depression, 15–19 moderately severe depression, and 20–27 severe depression (25)*.

b *Out of the 887 participants who responded to the AUDIT questionnaire, 858 (96.7%) completed the PHQ-9; 807 (91.0%) completed the GAD-7, and 772 (87.0%) completed the PSQI*.

c *Score of 0-4 was considered no GAD, 5-9 mild GAD, 10-14 moderate GAD, and 15-21 severe GAD*.

d *A score of 5 and above on the PSQI indicated poor quality sleep*.

### Prevalence of Harmful Alcohol Use

Three hundred and eighty nine (43.9%) participants reported harmful alcohol use based on an AUDIT score of 8 and above (95%CI: [40.6,47.2%]).

### Factors Associated With Harmful Alcohol Use

In bivariate analysis, gender, marital status, cadre and years of experience in the health field were significantly associated with harmful alcohol use (Tables 3, 4). In multivariate analysis, the factors significantly associated with increased odds of endorsing harmful alcohol use were: being male (AOR = 1.56; 95% CI = 1.14, 2.14; *p* = 0.006), being unmarried (AOR = 2.06; 95% CI = 1.48, 2.89; *p* < 0.001), having 11–20 years of experience in healthcare as compared to having 20+ years of experience (AOR = 1.91; 95% CI = 1.18, 3.12; *p* = 0.009), and being a specialist (AOR = 2.78; CI = 1.64, 4.78; *p* < 0.001) or doctor (AOR = 2.82; 95% CI = 1.74, 4.63; *p* < 0.001) or other cadre (AOR = 2.59; CI = 1.57,4.34; *p* < 0.001) as compared to being a nurse. Age, and endorsing depression or generalized anxiety disorder were not associated with harmful alcohol use (Table 5).

**Table 3:** Bivariate analysis of socio demographic factors and harmful alcohol use.

**Variable**		**Alcohol use** **(*****N*** **=** **887)**		***p*-value**
		**Harmful^**a**^** ***N* (%)**	**Not Harmful** ***N* (%)**	
Age in years	<35	198 (45.9)	233 (54.1)	0.251
	≥35	191 (41.9)	265 (58.1)	
Gender	Male	198 (49.1)	205 (50.9)	0.005
	Female	191 (39.5)	293 (60.5)	
Marital status	Married	224 (38.7)	355 (61.3)	<0.001
	Not married	165 (53.6)	143 (46.4)	
Years of experience in healthcare	0–10	238 (46.5)	274 (53.5)	0.001
	11–20	104 (47.5)	115 (52.5)	
	20+	47 (30.1)	109 (69.9)	
Cadre	Doctor	178 (50.3)	176 (49.7)	<0.001
	Nurse	38 (22.8)	129 (77.2)	
	Other	104 (48.1)	112 (51.9)	
	Specialist	68 (45.6)	81 (54.4)	
Type of facility	Public	265 (42.7)	356 (57.3)	0.312
	Private	124 (46.6)	142 (53.4)	
Have a known medical condition	Yes	97 (48.0)	105 (52.0)	0.202
	No	292 (42.6)	393 (57.4)	
Contact COVID-19 patients	Yes	92 (43.4)	120 (56.6)	0.940
	No	297 (44.0)	378 (56.0)	

a *Harmful alcohol use was defined by a score of 8 and above on the AUDIT*.

**Table 4:** Bivariate analysis of mental disorder and harmful alcohol use.

**Variable**		**Alcohol use** **(*****N*** **=** **887)**		***p*-value**
		**Harmful^**a**^** ***N* (%)**	**Not Harmful** ***N* (%)**	
Depression	Mild	241 (41.5)	340 (58.5)	0.065
	Moderate	63 (43.8)	81 (56.2)	
	Severe	70 (52.6)	63 (47.4)	
GAD	None/minimal	211 (40.9)	305 (59.1)	0.061
	Mild/Moderate	115 (49.6)	117 (50.4)	
	Severe	29 (49.2)	30 (50.8)	
PSQI	Poor quality sleep	86 (45.5)	103 (54.5)	0.672
	Good quality sleep	253 (43.4)	330 (56.6)	

a *Harmful alcohol use was defined by a score of 8 and above on the AUDIT*.

**Table 5:** Multivariate analysis of association between harmful alcohol use and socio-demographic and mental health factors.

**Characteristic**	**AOR^**a**^**	**95% CI^**b**^**	***p*-value**
**Age in years**			
<35	1		
≥35	1.10	0.70, 1.72	0.700
**Gender**			
Female	1		
Male	1.56	1.14, 2.14	0.006
**Marital status**			
Married	1		
Not married	2.06	1.48, 2.89	<0.001
**Years of experience in healthcare**			
20+	1		
11–20	1.91	1.18, 3.12	0.009
0-10	1.53	0.88, 2.69	0.140
**Cadre**			
Nurse	1		
Specialist	2.78	1.64, 4.78	<0.001
Doctor	2.82	1.74, 4.63	<0.001
Other	2.59	1.57, 4.34	<0.001
**PHQ**			
Mild	1		
Moderate	1.15	0.73, 1.81	0.500
Severe	1.50	0.90, 2.52	0.120
**GAD**			
None/minimal	1		
Mild/Moderate	1.07	0.72, 1.57	0.700
Severe	1.13	0.52, 2.44	0.800

a *Adjusted Odds Ratio*.

b *Confidence Interval*.

## Discussion

This cross-sectional study found that 43.9% of the healthcare workers in Kenya reported harmful patterns of alcohol use at the beginning of the COVID-19 pandemic in Kenya. Our findings are consistent with those reported by Hennein et al. (14) who found that 42.6% of healthcare workers in the US met criteria for probable alcohol use disorder, based on the AUDIT-Concise, during the COVID-19 pandemic. Much lower rates of harmful alcohol use (using the AUDIT-Concise) have been reported among healthcare workers in Europe i.e., 7% in the UK and 9.1% in Italy. Possible reasons for differences in rates include variations in alcohol control policies across regions during the pandemic (32) and disparities in the availability of treatment and prevention services for harmful alcohol use (33), emphasizing the need for context specific evidence. Another potential reason for varied findings could be related to differences in cut-off scores used to make a diagnosis. While Hennein et al. used a cut-off score of 4 or more, the studies conducted in the UK (15) (Greenberg) and Italy (16) used a cut-off score of >7.

The high rate of harmful alcohol use found in our setting in the early phase of the COVID-19 pandemic in Kenya is worrying. Firstly, such high rates threaten to reduce the capacity of the health workforce in Kenya to adequately respond to the pandemic. Secondly, consumption of alcohol use is expected to rise during the pandemic in Kenya. Studies conducted among healthcare workers in Europe and the United States (US) have reported increases in alcohol consumption among health providers as the number of COVID-19 cases rise and the preventive measures intensify (10–12). Thirdly, Kenya is already plagued by existing health worker shortages (3) and there is need to optimize the available human resources. We therefore call on the government through the relevant ministries to urgently put in place measures to mitigate the negative impact of harmful alcohol use on health service delivery during the pandemic.

In our study, being male was associated with increased odds of harmful alcohol use. This finding is consistent with prior studies conducted among healthcare workers (34) and the general population (35) in Kenya, and among healthcare workers during the COVID-19 pandemic in Italy (16). This might be explained by the fact that in many cultures, traditional gender roles, as well as strict cultural beliefs and values, may prevent the development of problematic substance use for women (36). In addition, men have been shown to have more opportunities to use substances like alcohol, as compared to women (37). Unmarried healthcare workers were more likely to report harmful alcohol use compared to the married. This is finding is consistent with other studies that have shown a higher prevalence of alcohol use among single or divorced persons (38). Being unmarried may be associated with social isolation, a welldocumented risk factor for harmful substance use (39, 40). Specialists, doctors and other cadres were significantly more likely to endorse harmful alcohol use as compared to nurses. A likely reason for this is that a majority of doctors in Kenya are male (and being male was associated with higher odds of endorsing harmful alcohol use in our study) while most nurses are female. In addition, nurses in Kenya have strong social support systems (e.g., they frequently turn to each other for emotional and practical support during times of distress), that could potentially prevent the use of alcohol as a way of coping with stress during the pandemic. Having 11–20 years of experience in the health profession was associated with increased odds of harmful alcohol use as compared to having 20+ years or having 0–10 years of experience. Findings concerning the association between years of experience and harmful alcohol use have been inconsistent. Obadeji et al. (41) in a study conducted among doctors in Nigeria reported no association between years of experience and hazardous alcohol use (42). Kenna and Lewis found alcohol use disorder among healthcare providers to be associated with having younger licensees (43). A possible reason for significant harmful alcohol use among healthcare workers in Kenya with 11–20 years of experience could be that that phase represents a period of heightened psychological stress linked to residency, and increasing family and work place responsibilities. Our study reported no significant differences in the rates of harmful alcohol use among healthcare workers with and without depression and generalized anxiety. This is inconsistent with studies showing alcohol use to be associated with mental health problems among healthcare workers during the pandemic (12, 17). Future longitudinal studies ought to shed more light on this finding.

### Implications for Practice

In order to optimize healthcare worker productivity during the COVID-19 pandemic in Kenya, it is important that interventions targeting harmful alcohol use are put in place for the at risk groups. Unmarried males, those working in doctor or specialist positions, and those with 11–20 years of experience were found to be at risk of harmful alcohol use during the pandemic. The Ministry of Health in partnership with the National Authority for the Campaign Against Alcohol and drug Abuse (NACADA) ought to implement programs for the at risk groups including: (i) routine screening and brief interventions for harmful alcohol use targeting the at risk groups (ii) health education on the harmful impact of alcohol use and debunking of myths that encourage alcohol use during the pandemic (ii) education on strategies for health promotion and self-care such as a healthy diet, adequate sleep, physical activity, and stress management to discourage use of alcohol as a way of coping (44, 45). The health education sessions and brief interventions should be tailored to be acceptable to, and focus on the needs of doctors and specialists, males, the unmarried and those with 11–20 years of experience.

Health care workers have professional associations that conduct regular continuous professional development sessions virtually. These form an avenue through which the education interventions outlined above may be delivered by fellow doctors. The Ministry of Health has established a call centre whose aim is to offer both knowledge and psychosocial support to frontline health workers (46). Brief interventions could be delivered by counselors or psychologists using this channel. At institutional level, facility heads should implement regular education sessions for staff and these ought to be done virtually in compliance with COVID-19 measures. Treatment and prevention interventions for harmful alcohol use among healthcare workers ought to be incorporated in national and institutional policies for managing the health workforce in Kenya during the pandemic in order to guide and encourage implementation.

Finally, it is important that mental health systems are strengthened overall. This way, mental health services will be accessible not only for the health care providers who need them, but for the entire population as well.

We acknowledge some limitations. Firstly, this being an online survey, it may have been less accessible to people who lacked internet access e.g., healthcare workers living in marginalized areas. Our findings may therefore not include their experiences. Secondly, we used a convenience sample hence the results may not be generalizable to other settings. Thirdly, this was a cross-sectional study and therefore no causal relationships may be determined. Fourthly, our sample was not representative of the composition of healthcare workers in Kenya. Our sample was comprised of mostly doctors while nurses comprise more than a half of healthcare workers in Kenya. Finally, we did not conduct sub-analyses by gender yet gender may have played an important role in explaining some associations. Nonetheless this study provides for important information on harmful alcohol use among healthcare workers at the beginning of the COVID-19 pandemic in a sub-Sahara African country.

## Conclusion

In conclusion, a high proportion of healthcare workers reported harmful alcohol use at the beginning of the COVID-19 pandemic in Kenya. Males, the unmarried, those with 11–20 years of experience in healthcare, and healthcare workers other than nurses, were more likely to report harmful alcohol use. Given the potential negative impact of harmful alcohol use not only on health service delivery but also on the mental and physical health of the healthcare workers, it is critical that the government puts in place interventions to address this problem. Specifically, we recommend that two key interventions be implemented (i) health education be done on the harmful effects of alcohol use and on strategies for promoting mental health (ii) screening and brief interventions for harmful alcohol use. Virtual platforms and mobile health strategies could be utilized to deliver these interventions in light of the COVID-19 preventive measures.

## Data Availability Statement

The datasets analyzed during the current study are available from the corresponding author on reasonable request.

## Ethics Statement

The studies involving human participants were reviewed and approved by Moi University/Moi Teaching Referral Hospital Institutional Research and Ethics Committee. The Ethics Committee waived the requirement of written informed consent for participation.

## Author Contributions

AM conducted the analysis. FJ drafted the manuscript. All authors participated in designing the study, contributed to and reviewed all versions of the manuscript, and approved the final version of the manuscript.

## Funding

This work was completed with support by Kenya Medical Association, Equity project.

## Conflict of Interest

The authors declare that the research was conducted in the absence of any commercial or financial relationships that could be construed as a potential conflict of interest.

## Publisher's Note

All claims expressed in this article are solely those of the authors and do not necessarily represent those of their affiliated organizations, or those of the publisher, the editors and the reviewers. Any product that may be evaluated in this article, or claim that may be made by its manufacturer, is not guaranteed or endorsed by the publisher.

## Footnotes

^1^Clinical officers are non-physician clinicians whose role is to provide physician-type health services in Kenya. This cadre was introduced due to a shortage in qualified doctors in the country (24).

^2^The main role of public health officers in Kenya is to plan preventive and promotive health programs.
